# Antioxidant, Antidiabetic, Anticholinergic, and Antiglaucoma Effects of Magnofluorine

**DOI:** 10.3390/molecules27185902

**Published:** 2022-09-11

**Authors:** Lokman Durmaz, Hatice Kiziltas, Leyla Guven, Hasan Karagecili, Saleh Alwasel, İlhami Gulcin

**Affiliations:** 1 Department of Medical Services and Technology, Cayirli Vocational School, Erzincan Binali Yildirim University, Erzincan 24500, Turkey; 2 Department of Pharmacy Services, Vocational School of Health Services, Van Yuzuncu Yil University, Van 65080, Turkey; 3 Department of Pharmaceutical Botany, Faculty of Pharmacy, Ataturk University, Erzurum 25240, Turkey; 4 Department of Nursing, Faculty of Health Science, Siirt University, Siirt 56100, Turkey; 5 Department of Zoology, College of Science, King Saud University, Riyadh 11362, Saudi Arabia; 6 Department of Chemistry, Faculty of Science, Ataturk University, Erzurum 25240, Turkey

**Keywords:** Magnofluorine, phenolic compound, butyrylcholinesterase, antioxidant activity, carbonic anhydrase, acetylcholinesterase, α-glycosidase

## Abstract

Magnofluorine, a secondary metabolite commonly found in various plants, has pharmacological potential; however, its antioxidant and enzyme inhibition effects have not been investigated. We investigated the antioxidant potential of Magnofluorine using bioanalytical assays with 2,2-azinobis (3-ethylbenzothiazoline-6-sulfonic acid) (ABTS^•+^), *N*,*N*-dimethyl-*p*-phenylenediamine dihydrochloride (DMPD^•+^), and 1,1-diphenyl-2-picrylhydrazyl (DPPH^•^) scavenging abilities and K_3_[Fe(CN)_6_] and Cu^2+^ reduction abilities. Further, we compared the effects of Magnofluorine and butylated hydroxytoluene (BHT), butylated hydroxyanisole (BHA), α-Tocopherol, and Trolox as positive antioxidant controls. According to the analysis results, Magnofluorine removed 1,1-diphenyl-2-picrylhydrazyl (DPPH) radicals with an IC_50_ value of 10.58 μg/mL. The IC_50_ values of BHA, BHT, Trolox, and α-Tocopherol were 10.10 μg/mL, 25.95 μg/mL, 7.059 μg/mL, and 11.31 μg/mL, respectively. Our results indicated that the DPPH· scavenging effect of Magnofluorine was similar to that of BHA, close to that of Trolox, and better than that of BHT and α-tocopherol. The inhibition effect of Magnofluorine was examined against enzymes, such as acetylcholinesterase (AChE), α-glycosidase, butyrylcholinesterase (BChE), and human carbonic anhydrase II (hCA II), which are linked to global disorders, such as diabetes, Alzheimer’s disease (AD), and glaucoma. Magnofluorine inhibited these metabolic enzymes with Ki values of 10.251.94, 5.991.79, 25.411.10, and 30.563.36 nM, respectively. Thus, Magnofluorine, which has been proven to be an antioxidant, antidiabetic, and anticholinergic in our study, can treat glaucoma. In addition, molecular docking was performed to understand the interactions between Magnofluorine and target enzymes BChE (D: 6T9P), hCA II (A:3HS4), AChE (B:4EY7), and α-glycosidase (C:5NN8). The results suggest that Magnofluorine may be an important compound in the transition from natural sources to industrial applications, especially new drugs.

## 1. Introduction

The oxidation of biological and food molecules leads to the creation of free radicals, resulting in the deterioration of cells and foods [1]. Methods such as air locking, temperature reduction, and the addition of antioxidant substances during processing, transportation, and storage are generally used to prevent oxidation of such components. The biological role of antioxidants is to neutralize cellular free radicals and reactive oxygen species (ROS) that have a negative effect on living organisms. Antioxidants terminate the oxidation of free radicals [2]. Antioxidant-rich molecules can be naturally created in humans or taken into the body exogenously with dietary components and food supplements [3]. However, the addition of antioxidants to the medium is the most convenient and economical method for processing oils and foods. What are these antioxidants? In terms of food, antioxidants are natural or synthetic substances that delay or completely prevent deterioration due to the autoxidation of fats and oils, even at low concentrations [4,5]. From a biological point of view, antioxidant compounds can protect the metabolism from the dangerous effects of oxidative stress and ROS [6,7]. Oxidative stress is a relatively new notion that has lately gained popularity in medicine and fundamental sciences [8]. Further, antioxidants prevent some chronic diseases, including diabetes mellitus (DM), cancer, cataract, and cardiovascular disorders [9,10]. They can neutralize free radicals and ROS and terminate damage to the biomolecules found in cells and tissues [11,12,13]. Although commercially used synthetic antioxidants have negligible toxicity at the prescribed doses, natural antioxidants have a higher demand and are safer [14,15,16].

Recently, it has been reported that antioxidants inhibit enzymes such as butyrylcholinesterase (BChE), acetylcholinesterase (AChE), α-amylase, carbonic anhydrase, and α-glycosidase, which are associated with diseases such as type 2-DM (T2DM), Alzheimer’s disease (AD), and glaucoma [15,16,17]. Thus, antioxidants can help treat the above-mentioned disorders [18]. Further, antioxidants play an important role in the prevention of T2DM and AD [19,20,21]. Some recent and basic scientific studies have revealed a link between T2DM and AD [22,23]. Therefore, AChE inhibitors (AChEIs) are known to cure AD. However, numerous drugs, including tacrine, have some hepatotoxic effects [24,25]. Among them, tacrine has some undesired effects, such as nausea, weight loss, vomiting, agitation, stomach upset, skin rash, chills, and diarrhea [26]. Thus, there is a need for the design and development of novel α-glycosidase and AChE inhibitors of natural origin [27,28].

Alkaloids are natural and active herbal metabolites containing one nitrogen atom in their chemical structure [29]. They are derived from benzylisoquinolines by removing two hydrogen atoms from two benzene nuclei, resulting in the formation of a 9,10-dihydrophenanthrene structure known as Magnofluorine, which is enzymatically synthesized in a few steps from dopamine and 4-hydroxyphenylacetate [30]. Among them, Magnofluorine is important from a biological point of view. Magnofluorine has multiple biological effects, such as anti-inflammatory, immunomodulatory, anticancer, antiallergic, cardiovascular, antibacterial, anti-osteoporotic, antiviral, and antifungal activities [31]. It can pass through the brain–blood barrier and affect the central nervous system. However, no study has investigated the antioxidant and enzyme inhibition effects of Magnofluorine.

AD is a rapidly progressing neurological illness with behavioral changes, forgetfulness, memory loss, and impaired language and cognition [32]. AChE decomposes acetylcholine (ACh) to acetate (CH_3_COO^−^) and choline (Ch), while BChE catalyzes the breakdown of butyrylcholine (BCh) to butyrate and Ch [33]. Of the two enzymes, AChE hydrolyzes several ChEs in the body, pancreas, blood, CNS, and liver [34], and it is found in erythrocyte membranes, nerves, muscle, CNS, peripheral tissues, and cholinergic, non-cholinergic, sensory, and motor fibers. In addition, BChE is mainly related to the glial and endothelial cells in the brain [33]. Many recent studies have examined the nature of AChE inhibition to elucidate the effect of ACh receptor binding [35]. However, the exact physiological role of another cholinergic enzyme, BChE, has not been completely elucidated. When AChE activity begins to decline during AD progression, BChE has been shown to functionally complement this enzyme and play a prominent role. However, it is well-known that selective inhibitors of the enzymes are important in designing active drugs against neurodegeneration, and they play an important part in AD treatment. Therefore, dual co-inhibitors of AChE/BChE enzymes can be a promising therapeutic alternative to treat AD [36].

Carbonic anhydrases (CA) are metalloenzymes, which catalyze water and carbon dioxide (CO_2_) to reversibly hydrate into protons (H^+^) and bicarbonate (HCO_3_) and metabolize acid-base concentrations having Zn^2+^ in their active side structures [37,38]. Additionally, they maintain fluid equilibrium throughout the body, especially in the eyes, stomach, and kidneys. The high intraocular pressure (IOP) associated with glaucoma can be alleviated or treated using carbonic anhydrase inhibitors (CAIs) [39,40]. Thus, hCA II inhibition is a critical mechanism of action in treating glaucoma-related IOP reduction [41]. It is well-known that glaucoma is one of the main causes of blindness worldwide. It is also conjectured that the number of people suffering from glaucoma worldwide will reach 120 million by 2040 [36,42]. Clinically, laser, pharmacological therapies, and surgery are the main treatment methods for glaucoma. Thus, it is important to use CAIs topically to prevent their undesired effects.

We conducted this study to investigate the antioxidant abilities of Magnofluorine using bioassays such as Fe^3+^ and Cu^2+^ reduction, DMPD^•+^, ABTS^•+^, and DPPH^•^ scavenging abilities and test the purposed inhibitory abilities of Magnofluorine towards BChE, hCA II, AChE, and α-glycosidase associated with glaucoma, AD, and T2DM diseases.

## 2. Results

**Figure 1:**
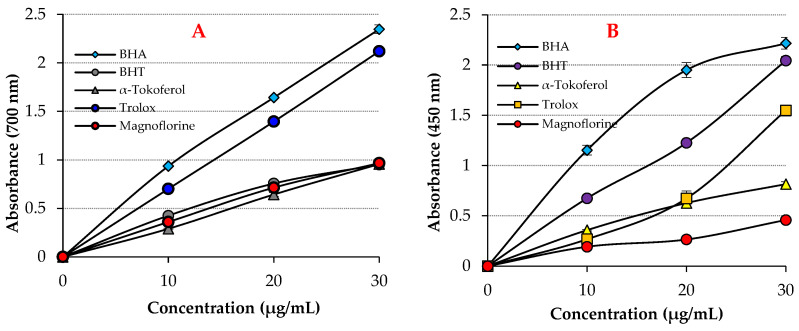
Fe^3+^ (**A**) and Cu^2+^ (**B**) ions reducing abilities of Magnofluorine and standards.

A variety of methodologies and activity assessments are used to prevent oxidation using antioxidants. As seen in Figure 1A and Table 1, Magnofluorine showed effective Fe^3+^-reducing ability (*p* < 0.01). The reducing effect of Magnofluorine and the positive controls increased in a concentration-dependent manner (30 μg/mL) for the tested materials. The Fe^3+^ reducing ability of Magnofluorine and the positive controls decreased as follows: BHA (λ_700_: 2.347, r^2^: 0.9086) > Trolox (λ_700_: 2.119, r^2^: 0.9586) > Magnofluorine (λ_700_: 0.967, r^2^: 0.9938) > α-Tocopherol (λ_700_: 0.957, r^2^: 0.9863) ≥ BHT (λ_700_: 0.952, r^2^: 0.9154). The results demonstrated that the Fe^3+^ reduction ability of Magnofluorine is better than that of BHT and α-Tocopherol, but lower than that of Trolox and BHA. Additionally, previous studies have reported the Fe^3+^-reducing absorbance values to be 0.278 (r^2^: 0.9567) [12], 2.769 (r^2^: 0.9945) [43], 0.739 (r^2^: 0.9778) [3], 0.432 (r^2^: 0.9981) [44], 2.509 (r^2^: 0.9906) [45], and 2.428 (r^2^: 0.9474) [46] for similar quantities of usnic acid, caffeic acid, coumestrol, uric acid, CAPE, and tannic acid, respectively.

The Cu^2+^ reduction ability of Magnofluorine and the positive controls (30 μg/mL) is shown in Figure 1B and Table 1. Further, the Cu^2+^-reducing ability of Magnofluorine was dose-dependent (10–30 μg/mL). The Cu^2+^-reducing ability of Magnofluorine and the positive controls decreased as follows: BHA (λ_450_: 2.216, r^2^: 0.9928) > BHT (λ_450_: 2.044, r^2^: 0.9937) Trolox (λ_450_: 1.548, r^2^: 0.9305) > α-Tocopherol (λ_450_: 0.816, r^2^: 0.9897) > Magnofluorine (λ_450_: 0.458, r^2^: 0.9938). Additionally, the absorbance values of the same concentration of natural phenolics such as usnic acid, coumestrol, resveratrol, eugenol, olivetol, and taxifolin are 0.277 (r^2^: 0.9836) [12], 0.739 (r^2^: 0.9778) [3], 0.085 (r^2^: 0.8403) [47], 0.762 (r^2^: 0.9957) [48], 1.314 (r^2^: 0.9682) [49], and 0.750 (r^2^: 0.9550), respectively [50].

**Figure 2:**
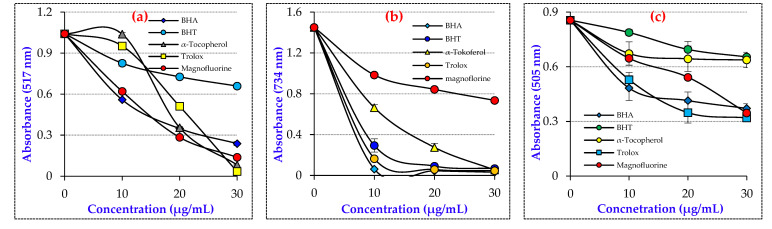
Radical scavenging effects of Magnofluorine and positive controls. (**a**). DPPH^•^ scavenging ability, (**b**). ABTS^•+^ scavenging ability, (**c**). DMPD^•+^ scavenging ability.

The radical scavenging ability of Magnofluorine was determined using the antioxidant assays, DPPH^•^, ABTS^•+^, and DMPD^•+^ methods. Magnofluorine exhibited considerable DPPH^•^, ABTS^•+^, and DMPD^•+^ scavenging activities. The results exhibited that Magnofluorine significantly scavenged the DPPH radical in a concentration-dependent manner (10–30 μg/mL). In the DPPH^•^ removal studies, the IC_50_ value for Magnofluorine was found to be 10.58 μg/mL (r^2^: 0.9908) (Table 2 and Figure 2A). In contrast, the IC_50_ values were calculated as 7.059 μg/mL (r^2^: 0.9614), 10.10 μg/mL (r^2^: 0.9015), 11.31 μg/mL (r^2^: 0.9642), and 25.95 μg/mL (r^2^: 0.9221) for Trolox, BHA, α-tocopherol, and BHT, respectively. In this context, the IC_50_ value was calculated to be 3.30 μg/mL [45], 6.96 μg/mL [47], 16.06 μg/mL [48], 17.77 μg/mL [49], 20.0 mg/mL [51], 30.6 μg/mL [52], 34.9 μg/mL [53], 49.50 μg/mL [12], and 77.00 μg/mL [50] for CAPE, resveratrol, eugenol, olivetol, silymarin, L-Adrenaline, curcumin, usnic acid, and taxifolin, respectively.

ABTS radicals have higher reactivity than DPPH radicals. As seen in Table 2 and Figure 2B, Magnofluorine effectively eliminated ABTS radicals (IC_50_: 27.61 μg/mL, r^2^: 0.9006). Additionally, the IC_50_ values of 5.07 μg/mL for BHA (r^2^: 0.9356), 6.16 μg/mL for Trolox (r^2^: 0.9692), 6.99 μg/mL for BHT (r^2^: 0.9350), and 8.73 μg/mL for α-tocopherol (r^2^: 0.9015), were reported. The results exhibited that Magnofluorine had lower ABTS radical scavenging effects when compared to all of the positive controls as standard antioxidants.

**Figure 3:**
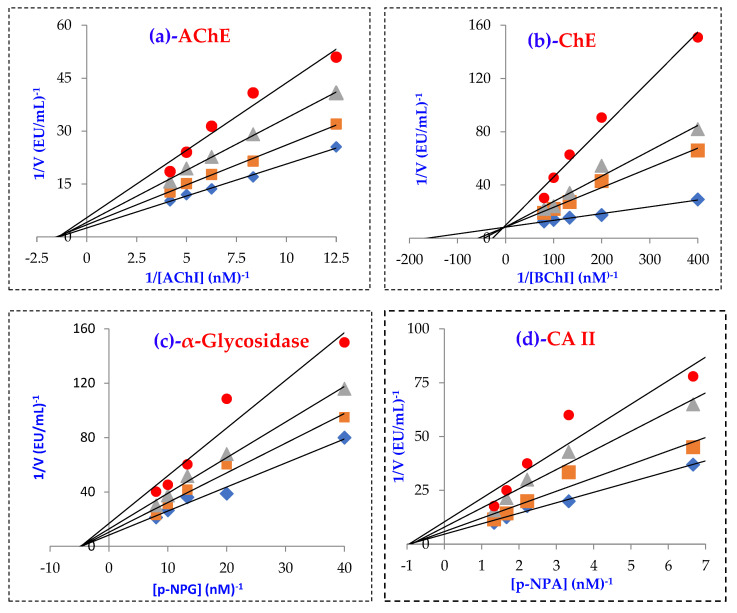
Lineweaver–Burk graphs of Magnofluorine towards acetylcholinesterase (AChE) enzyme (**a**), butyrylcholinesterase (BChE) enzyme (**b**), carbonic anhydrase II isoenzyme (CA II) (**c**), and α-glycosidase (**d**).

As seen in Table 2 and Figure 3C, the IC_50_ value for the DMPD^•+^ removal of Magnofluorine (IC_50_: 15.26 μg/mL, r^2^: 0.9966) had lower DMPD^•+^ removal than that of Trolox (IC_50_: 4.33 μg/mL, r^2^: 0.9447), α-tocopherol (IC_50_: 7.11 μg/mL, r^2^: 0.9509), BHT (IC_50_: 8.72 μg/mL, r^2^: 0.9375), and BHA (IC_50_: 11.99 μg/mL, r^2^: 0.9580), which were utilized as reference antioxidants in antioxidant studies. Lower IC_50_ values indicate higher DMPD^•+^ removal effects.

According to the enzyme inhibition results (Table 3), Magnofluorine effectively inhibited the cholinergic enzymes AChE and BChE with Ki values of 10.25 ± 1.94 and 2.47 ± 0.70 nM, respectively (Table 3 and Figure 3A,B). Additionally, Tacrine, as a clinical drug, had a K_i_ value of 5.99 ± 1.79 nM (Figure 3B) and 2.43 ± 0.92 nM for BChE and AChE (Figure 3A), respectively.

Furthermore, Magnofluorine had an efficient inhibition profile against the α-glycosidase as a proteolytic enzyme with a K_i_ value of 30.56 ± 3.36 nM (Table 3 and Figure 3c). It was reported that Acarbose exhibited an α-glycosidase enzyme with an IC_50_ value of 22,800 nM [54]. As seen in Table 3 and Figure 3d, Magnofluorine demonstrated a potent CA II inhibition effect (K_i_: 25.41 ± 1.10 nM) when compared to Acetazolamide (K_i_: 4.41 ± 0.35 nM) as a strong and clinical CA II inhibitor.

**Figure 4:**
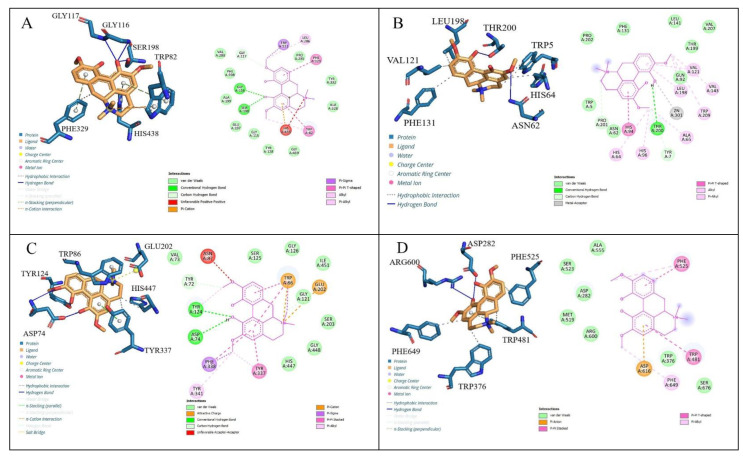
(**A**). The 2D and 3D interactions of BChE (6T9P) and Magnofluorine, (**B**). The 2D and 3D interactions of Carbonic anhydrase (II) enzyme (3HS4) and Magnofluorine, (**C**). The 2D and 3D interactions of AChE enzyme (4EY7) and Magnofluorine, (**D**). The 2D and 3D interactions of α-glycosidase enzyme (5NN8) and Magnofluorine.

According to the docking scores, Magnofluorine molecules effectively inhibited all enzymes, and these results were found to be compatible with the results of in vitro studies of this research. The molecular interactions of Magnofluorine with hCA II (A:3HS4), AChE (B:4EY7), BChE (D: 6T9P), and α-glycosidase (C:5NN8) are given in Table 4 and Figure 4.

## 3. Discussion

Antioxidants, even when found at extremely low concentrations in foods or the human body, can delay, limit, or completely block the oxidative processes, thus, enhancing food quality [55,56]. Of these, the most crucial function of an antioxidant molecule is reduction, shown by its electron-withdrawing capacity [57,58,59]. Antioxidant molecules scavenge free radicals and ROS by donating electrons and reducing themselves. Antioxidants can bind free radicals and protect biomaterials against oxidation, which are widely used to delay or prevent food oxidation [60]. The effectiveness of antioxidant compounds such as Magnofluorine depends on several factors, including temperature, structural features, sensitive substrate oxidation, concentration, presence of synergistic and pro-oxidants, and physical conditions [61]. Figure 1 and Figure 2 and Table 1 and Table 2 depict the antioxidant activities of Magnofluorine, which were determined using different chemical-based methodologies. The most putative ones are reducing abilities [62]. So far, different chemical assays combined with extremely sensitive and automated detection technologies have been utilized for the evaluation of antioxidant activity using unique approaches, including radical scavenging activity, metal chelation, and reduction potential. Reduction ability directly measures the transfer of electrons from an antioxidant to free radicals or transfer of hydrogen atoms. Additionally, the reduction potential of an antioxidant can be measured using several bioassays [63]. The addition of Magnofluorine to the Fe^3+^ solution enhances the formation of Prussian blue Fe_4_[Fe(CN)_6_], which has a maximum absorbance at 700 nm [64]. Therefore, the determination of high absorbance at this wavelength indirectly reflects the reducing ability of antioxidant molecules. The simplified Fe^3+^ reducing ability mechanism of Magnofluorine is illustrated as follows:

(1) $$\mathrm{Magnofluorine}+{\mathrm{Fe}}^{3+}\to \mathrm{Oxidized}\;\mathrm{Magnofluorine}+{\mathrm{Fe}}^{2+}$$

(2) $${\mathrm{Fe}}^{2+}+{\left[\mathrm{Fe}{\left(\mathrm{CN}\right)}_{6}\right]}^{3-}\to \mathrm{Fe}{\left[\mathrm{Fe}{\left(\mathrm{CN}\right)}_{6}\right]}^{-}$$

As shown in Table 1 and Figure 2A, the reducing activity increased with the increase in Magnofluorine concentration (μg/mL). In Magnofluorine reduction, ([Fe[(CN)_6_]^3−^) complex under the action of ferric trichloride is easily reduced to (Fe[Fe(CN)_6_]^−^) [65]. Higher the absorbance value, the better the reducing ability. Magnofluorine promises antioxidant activity owing to the hydroxyl groups (-OH) linked to aromatic rings in its backbone [66,67]. It was observed that Magnofluorine had a higher reducing ability as compared to standard molecules. The Cu^2+^ reducing assay (CUPRAC assay), which was devised in the early 2000s, has been used to determine the antioxidant ability of pure molecules [68]; however, it has already been changed for different assays for determining the antioxidant ability based on reduction of cupric (Cu^2+^) to cuprous ions (Cu^+^). Similar to other methods, this method uses a ligand to form a copper–ligand complex to measure absorbance. The ligand used for this purpose is the Neocuproine (2,9-dimethyl-1,10-phenanthroline) complex [69]. In the reduction experiment, reactive aromatic -OH groups of polyphenols such as Magnofluorine were oxidized to the corresponding quinones and reduced to Cu^2+^-Neocuproine. In this way, the reduced Cu^+^-Neocuproine complex with an intense yellow–orange color is formed [70].

**Figure 5:**
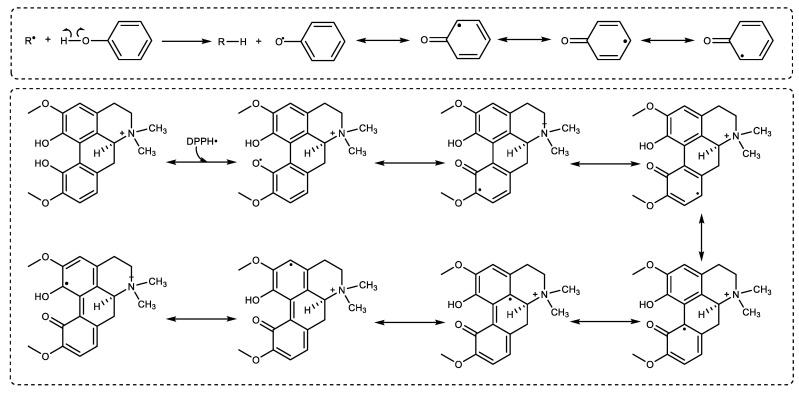
Proposed possible DPPH radical scavenging mechanism of Magnofluorine.

The radical scavenging activities of Magnofluorine were assessed with DMPD^•+^, ABTS^•+^, and DPPH^•^ radical scavenging activities, which were developed on different approaches providing evidence about free radicals and antioxidant agents [71]. Thus, the use of radical removing activity is quite easy for determining the antioxidant abilities of compounds [72]. The results exhibited that Magnofluorine had a similar DPPH· removing activity as compared to α-Tocopherol and BHA, but lover than Trolox and better than BHT. The results showed that Magnofluorine had a more effective DPPH free-radical-scavenging ability. In the DPPH test, the reduction of stable radical DPPH to yellow DPPH_2_ by Magnofluorine is used to measure the antioxidant ability of a molecule to act as a hydrogen atom or electron donor [73]. In light of this information, the possible mechanisms of Magnofluorine and DPPH radicals are illustrated in Figure 5 and the structure of Magnofluorine. 

Following the interaction of Magnofluorine and DPPH·, the radicals disappear after accepting an electron (e^−^) or hydrogen radical (H·) from Magnofluorine to become DPPH_2_ based on an electron transfer reaction [74]. The mechanism of the DPPH radical scavenging of Magnofluorine has not been previously reported. Additionally, the best information on this subject is that the radicals generated from the phenolic groups in Magnofluorine are stabilized due to resonance structures. In this way, a Magnofluorine molecule scavenges the two DPPH radicals and switches to a diketonic structure, and these radicals also switch to the neutral form. For comparison, the IC_50_ values (μg/mL) were calculated to be 0.83 [50], 1.94 [49], 25.95 [3] 6.93 [52], 6.96 [47], 7.84 [48], 8.62 [51], 9.80 [45], 10.41 [12], and 18.07 [53] for taxifolin, olivetol, coumestrol, L-Adrenaline, resveratrol, eugenol, silymarin, CAPE, usnic acid, and curcumin, respectively.

Antioxidants reduce the blue–green color of ABTS^•+^; this reaction was then followed by absorbance measurement at 734 nm. Another radical elimination method used in this study was the DMPD^•+^ scavenging ability. In this antioxidant method, antioxidant molecules transfer an H atom to DMPD radicals, removing the existing color and causing the solution to lighten [75]. The IC_50_ value for the DMPD^•+^ removing ability was more effective as compared to that reported previously. For instance, the IC_50_ (μg/mL) values were 9.5 [47], 10.04 [48], 12.81 [3], 15.6 [53] 19.25 [49], 26.70 [48], 33.00 [12], 34.5 [53], and 173.25 [50] for resveratrol, eugenol, coumestrol, L-Adrenaline, olivetol, CAPE, usnic acid, curcumin, and taxifolin, respectively.

Further, Magnofluorine was more than two times more effective than Tacrine against AChE, and it had a similar inhibition ability agisnt Tacrine against BChE. On examining the results, we observed that the selectivity index (AChE/BChE; 1.15) was in favor of AChE. In some recent clinical studies, some putative inhibitors of AChE, including rivastigmine, donepezil, and tacrine, have been used in the early stages of AD treatment. Tacrine was later clinically proven to have hepatotoxicity. Therefore, although tacrine is effective in the treatment of AD, it has been withdrawn from clinical trials [66]. Additionally, the K_i_ valuew of some molecules for AChE inhibition were calculated as 3.39 nM [12], 23.80 [3], 0.518, and 0.322 nM for usnic acid, coumestrol, and CAPE [40]. Moreover, K_i_ values (μg/mL) for AChE were recorded as 5.13 [49] and 16.70 [39] for olivetol and taxifolin, respectively.

T2DM is a common metabolic disorder originating from high blood glucose levels. Therefore, recent studies have focused on the inhibition of α-glucosidase that controls carbohydrate digestion [76]. Magnofluorine had a K_i_ value of 30.56 ± 3.36 nM towards α-glycosidase (Table 3 and Figure 3C). The results clearly exhibited that Magnofluorine had efficient α-glycosidase inhibition ability as compared to acarbose (IC_50_: 22,800 nM) [54]. The results also demonstrated that Magnofluorine had a more efficient Ki value as compared to acarbose as an effective starch blocker [77].

Since phenolic compounds have slightly acidic properties, they lose protons (H^+^) from their hydroxyl groups and form highly soluble phenolate anions in water. It is known that phenolic compounds can efficiently inhibit CA isoenzymes due to the presence of functional groups such as phenolic -OH, -OCH_3_, and -COOH groups in their scaffolds [78]. They inhibit CA II isozymes that coordinate to Zn^2+^ in the active cavity of CA. CA II isoform is involved in the protection of body fluids [79]. As shown in Table 3 and Figure 3D, when profiling the assay against cytosolic and predominant hCA II isoform, Magnofluorine had a Ki value of 25.41 ± 1.10 nM. In comparison, AZA demonstrated a Ki value of 4.41 ± 0.35 nM against cytosolic and dominant hCA II isoenzyme, which exist everywhere in the cells and tissues [80].

Docking studies were followed by an analysis of the binding modes to understand the inhibition mechanisms. According to docking scores, Magnoflorine exhibited high binding affinity with all enzyme targets (Figure 4 and Table 4). The binding affinity of the Magnoflorine-BChE (6T9P) complex was calculated to be −9.8 kcal/mol (Table 4). Magnoflorine formed three hydrogen bonds with Gly116, Gly117, and Ser198 residues in the active site of the BChE. Furthermore, the Magnoflorine-BChE complex showed hydrophobic interactions with Trp82 and Phe329 residues, π-π stacked interactions with Trp82 and Phe329, π cation interaction with His438, and π sigma interaction with Trp231 (Figure 4a). The binding affinity of the Magnoflorine-hCA II (A:3HS4) complex was calculated to be −8.2 kcal/mol (Figure 4b). Magnoflorine was shown to bind to the active site via two H-bond interactions between Asn62 and Thr200. Hydrophobic interactions with Trp5, His64, His96, Val121, Val143, Leu198, Phe131, π-π stacked interactions with Tyr337 and Phe338, and also the π-π T-shaped interaction with His94 were formed (Figure 4b).

Magnoflorine was placed in the active site of the enzyme AChE (PDB code: 4EY7). Figure 4c represents the 3D and 2D interactions of Magnoflorine-AChE, and the docking score was calculated to be −9.5 kcal/mol (Table 4). Magnoflorine was shown to bind to the active site via two H-bond interactions between its hydroxyl groups and the active site amino acids Tyr124 and Asp74. Moreover, hydrophobic contacts with Trp86; Tyr337; His447, π-π stacked interactions with Tyr337; Phe338, and also the π cation interaction with Trp86 were formed (Figure 4c). Magnoflorine-α-glycosidase (5NN8) complex’s docking score was calculated as −7.2 kcal/mol (Table 4). The interactions in the Magnofluorine binding mode comprised two H bonds with Arg600 and Asp282 active site amino acids, four hydrophobic interactions with Trp481, Trp376, Phe525, Phe649, and a π anion interaction with Asp616 (Figure 4d). According to the docking scores, it has been determined that the Magnoflorine effectively inhibited all of the studied enzymes, and these results were found to be compatible with the results of in vitro studies of this research.

## 4. Materials and Methods

### 4.1. Chemicals

Magnofluorine (≥98% (HPLC)), acetylcholinesterase, butyrylcholinesterase, α-glycosidase, *p*-nitrophenyl-*D*-glucopyranoside, α-tocopherol, acetylcholine iodide, butyrylcholine iodide, 2,2-azinobis (3-ethylbenzothiazoline-6-sulfonic acid), *N*,*N*-dimethyl-*p*-phenylenediamine dihydrochloride and 1,1-diphenyl-2-picrylhydrazyl were obtained from Sigma-Aldrich GmbH (Steinheim, Germany). For the antioxidant activity, Magnofluorine was dissolved in ethanol; however, for enzyme inhibition studies, Magnofluorine was dissolved in DMSO due to the possible inhibition effects of ethanol.

### 4.2. Antioxidant Assays

The Fe^3+^ reducing ability of Magnofluorine was investigated and compared with the reduction abilities of the positive controls. Different concentrations of dissolved Magnofluorine (10–30 μg/mL) were mixed with 2 mL of sodium phosphate buffer (200 mM, pH 6.6) and 1 mL K_3_Fe(CN)_6_ (1%), and the solution was incubated at 50 °C for 25 min. The reaction was terminated by adding 1 mL of trichloroacetic acid (TCA, 10%). Following this, 0.5 mL of newly prepared FeCl_3_ (0.1%) was transferred, and the absorptions were measured at 700 nm. Deionized water was used as the blank control. The Cu^2+^ reduction ability of Magnofluorine was realized at 450 nm according to previous procedures [81]. Magnofluorine was prepared at diverse concentrations (10–30 μg/mL) and added to 250 μL CuCl_2_ solution (10 mM), 250 μL neocuproine solution prepared in ethanol (7.5 mM), and 250 μL of acetate buffer (1.0 M). Finally, after 20 min, absorbances were recorded at 450 nm [68].

The DPPH radical scavenging activity of Magnofluorine was estimated according to the Blois method at 517 nm [82]. Briefly, 0.2 mL of Magnofluorine in different concentrations of ethyl alcohol (10–30 μg/mL), 0.2 mL of DPPH solution (0.3 mM) in methanol, and 0.6 mL ethanol were added to test tubes, and the tubes were incubated at 37 °C for 30 min. The DPPH radical scavenging activities were assessed by measuring the absorbance at 517 nm after keeping in the dark for 30 min [81]. Further, the ABTS radical scavenging activity of Magnofluorine was determined. ABTS solution (7.0 mM) was prepared using K_2_S_2_O_8_ (2.45 nM), and the absorbance of the control was set to 0.700 ± 0.025 at 734 nm on dilution with buffer solution (0.1 M and pH 7.4). Finally, 1 mL of ABTS radicals was transferred to different concentrations of Magnofluorine (10–30 μg/mL), and the absorbance was recorded at 734 nm. The control included only ABTS radical solution [82]. The DMPD radical removing ability of Magnofluorine was determined at 505 nm according to a previous method [83]. Briefly, 0.2 mL of FeCl_3_ (50 mM) and 1 mL of DMPD solution were added to the buffer (pH 5.3, 100 mM). The concentrations of all of the samples were 10–30 μg/mL. The total volume was adjusted to 0.5 mL using deionized water. An aliquot (1 mL) of DMPD radicals was added, and the absorbance was recorded at 505 nm. The radical scavenging results were expressed as half maximal scavenging concentrations (IC_50_, μg/mL) [84].

### 4.3. Anticholinergic Assay

The inhibition effect of Magnofluorine on AChE from *Electrophorus electricus* was realized according to a previous study [85]. AChI and BChI were used as substrates for enzymatic reactions. An aliquot (0.1 mL) of Tris/HCl buffer (pH 8.0, 1.0 M) and different concentrations of Magnofluorine (10–30 μg/mL) were added to 50 μL of the AChE/BChE solution (5.30 × 10^−3^ EU), and the mixture was incubated at 20 °C for 20 min. Following this, 50 μL of 5,5′-dithio-bis 2-nitro-benzoic acid (DTNB, 0.5 mM) and achethylcholine iodade (AChI)/butyrylcholine iodade (BChI) were added and enzymatic reactions were initiated. The AChE/BChE activities were spectrophotometrically determined at 412 nm [62].

### 4.4. Antidiabetic Assay

The inhibitory effect of Magnofluorine against α-Glycosidase was determined according to the methods described by Tao et al. [54] and Hashmi et al. [86] using *p*-nitrophenyl-D-glucopyranoside (*p*-NPG) as the substrate. Firstly, 75 μL of phosphate buffer (pH 7.4) was mixed with 5 μL of sample and 20 μL α-glycosidase enzyme solution (0.15 U/mL) in phosphate buffer (pH 7.4). Following a short period of incubation (10 min), 50 μL of *p*-nitrophenyl-D-glycopyranoside (*p*-NPG) in phosphate buffer (5 mM, pH 7.4) was added and incubated at 37 °C, and absorbance was measured at 405 nm [87]. One unit is the quantity of α-glycosidase, which hydrolyzes 1.0 mol substrate per minute (pH 7.4) [88].

The α-amylase inhibition effects of Magnofluorine were determined as described previously [89]. Principally, 1 g starch was dissolved in 50 mL NaOH solution (0.4 M) and heated at 80 °C for 20 min. After cooling, the pH was adjusted to 6.9, and the volume was adjusted to 100 mL using distilled water. Next, 35 μL of starch solution, 35 μL of phosphate buffer (pH 6.9), and 5 μL of the Magnofluorine solutions were mixed. After incubation at 37 °C for 20 min, 20 μL of enzyme solution was added and incubated again for 20 min. The reaction was completed by adding 50 μL of 0.1 M HCl, and absorbance was measured at 580 nm.

### 4.5. Antiglaucoma Assay

The CA II isozyme was purified from human erythrocytes using Sepharose-4B-Tirozyne-sulfanylamide affinity column chromatography [90]. Further, it was precipitated, and the serum was separated and adjusted with solid Tris to pH 8.7. The sample was then loaded to the affinity chromatography column and equilibrated with Tris-Na_2_SO_4_/HCl (pH 8.7, 22 mM/25 mM). CA II was eluted with sodium acetate/NaClO_4_ (0.5 M, pH 5.6, 25 °C) [91]. Protein quantity during the purification study was determined using the Bradford method [92]. Bovine serum albumin was used as the standard protein [93]. The purity of CA II was controlled using SDS-PAGE [94]. During purification and inhibition of CA II, esterase activities were performed following the change in absorbance at 348 nm [95]

### 4.6. Inhibition Parameters

The IC_50_ values were calculated from activity (%) versus Magnofluorine plots [96]. First, the enzyme inhibitions were studied at different Magnofluorine concentrations. The obtained values were plotted as % activity against Magnofluorine concentrations. Then, the Magnofluorine concentrations, which cause 50% enzyme inhibition (IC_50_), were calculated from these graphs. The K_i_ values and other parameters were calculated from Lineweaver-Burk graphs [97] as described priorly [98]. The K_i_ values were taken out from this graph [75]. All of the analyses were independently conducted in triplicate, and the results are expressed as mean values ± SD.

### 4.7. Molecular Docking Studies 

The crystal structures of the AChE (PDB ID:4EY7) [99], α-glycosidase (PDB ID:5NN8) [100], BchE (PDB ID:6T9P) [101], and hCA II (PDB ID:3HS4) [102] enzymes were downloaded from the “Protein Data Bank” website with resolutions of 2.35 Å, 2.45 Å, 2.70 Å, and 1.10 Å, respectively [103]. The structures of these enzymes were optimized in AutoDock-Tools 1.5.7 [104]. The 3D version of the chemical structure of Magnofluorine was downloaded from the pubChem database (https://pubchem.ncbi.nlm.nih.gov/ accessed on 2 August 2022). Structure optimization and the most stable conformations of the ligands were determined with AutoDockTools. Then, the PDBQT file of the ligands was prepared. AutoDock-Tools program was used for docking. The binding interactions were analyzed with BIOVIA Discovery Studio Visualizer v21.1.0.20298 (Accelrys Software Inc., San Diego, CA, USA, 2012, https://pubchem.ncbi.nlm.nih.gov/ accessed on 2 August 2022) and PLIP [105].

### 4.8. Statistical Analysis 

Statistical analyses were performed by unpaired Student’s *t*-test using the statistical program of IBM SPSS Statistics 20. The results were recorded as means with their standard deviation (SD). *p* < 0.05 was the minimum significance level.

## 5. Conclusions

In this study, Magnofluorine exhibited an efficient antioxidant profile as compared to the standards including BHA, BHT, α-Tocopherol, and Trolox. Furthermore, Magnofluorine, which possesses a wide spectrum of biological activities, was found to neutralize ROS and free radicals by donating a hydrogen atom or electron to free radicals. The results obtained from this study showed that Magnofluorine, a safer natural phenolic antioxidant, can be used to prevent or delay the formation of lipid autoxidation. In this way, it can extend the shelf-life of materials processed in the pharmaceutical and food industries and maintain their nutritional quality for a long time. Additionally, Magnofluorine was tested against some metabolic enzymes, including acetylcholinesterase, α-glycosidase, butyrylcholinesterase, and carbonic anhydrase isoform II, which are linked to some common and global diseases, such as epilepsy, diabetes, Alzheimer’s disease, and glaucoma. Finally, the results contributed to the evidence that Magnofluorine has biological effects such as anticholinergic, antidiabetic, and antiglaucoma effects. Thus, it can be used in the treatment of diseases after approval by in vivo and clinical studies.

## Figures and Tables

**Table 1:** Fe^3+^ and Cu^2+^ reduction abilities of Magnofluorine and positive controls at 30 μg/mL concentration.

Antioxidants	Fe^3+^ Reducing		Cu^2+^ Reducing	
	λ (700 nm)	r^2^	λ (450 nm)	r^2^
**BHA**	2.347	0.9086	2.216	0.9928
**BHT**	0.952	0.9154	2.044	0.9937
**Trolox**	2.119	0.9586	1.548	0.9305
**α-Tocopherol**	0.957	0.9863	0.816	0.9897
**Magnofluorine**	0.967	0.9938	0.458	0.9729

**Table 2:** IC_50_ (μg/mL) values for DPPH^•^, DMPD^•+^, and ABTS^•+^ scavenging of Magnofluorine and standard antioxidants.

Antioxidants	DPPH^•^ Scavenging		ABTS^•+^ Scavenging		DMPD^•+^ Scavenging	
	IC_50_	r^2^	IC_50_	r^2^	IC_50_	r^2^
**BHA**	10.10	0.9015	5.07	0.9356	11.99	0.9580
**BHT**	25.95	0.9221	6.99	0.9350	8.72	0.9375
**Trolox**	7.05	0.9614	6.16	0.9692	4.33	0.9447
**α-Tocopherol**	11.31	0.9642	8.37	0.9015	7.11	0.9509
**Magnofluorine**	10.58	0.9908	27.61	0.9006	15.16	0.9966

**Table 3:** Inhibition values of Magnofluorine against α-glycosidase (α-Gly), carbonic anhydrase isoenzyme II (CA II), butyrylcholinesterase (BChE), and acetylcholinesterase (AChE) enzymes.

Compounds	IC_50_ (nM)								K_i_ (nM)			
	CA II	r^2^	AChE	r^2^	BChE	r^2^	α-Gly	r^2^	CA II	AChE	BChE	α-Gly
**Magnofluorine**	26.03	0.9313	10.01	0.9429	8.71	0.9825	31.02	0.9364	25.41 ± 1.10	10.25 ± 1.94	2.47 ± 0.70	30.56 ± 3.36
Acetazolamide *	8.37	0.9825	-	-	-	-	-	-	4.41 ± 0.35	-	-	-
Tacrine **	-	-	5.97	0.9706	8.37	0.9846	-	-	-	2.43 ± 0.92	5.99 ± 1.79	-
Acarbose ***	-	-	-	-	-	-	22,800	-	-	-	-	-

* Acetazolamide (AZA) is a standard for CA II inhibition. ** Tacrine (TAC) is a standard for AChE inhibition. *** Acarbose (ACR) is a standard for α-glycosidase inhibition [54].

**Table 4:** Molecular interactions of Magnofluorine with α-glycosidase (α-Gly, C:5NN8), human carbonic anhydrase isoenzyme II (hCA II, A:3HS4), butyrylcholinesterase (BChE, D: 6T9P), and acetylcholinesterase (AChE, B:4EY7) enzymes.

Complex	Docking Scores (kcal/mol)	Types of Interactions	Interacting Residues
**hCA II (3HS4)-Magnofluorine**	−8.2	Hydrogen bonding Hydrophobic interactions π-π T-shaped	Asn62, Thr200, Trp5; His64, His96, Val121, Val143, Leu198, Phe131, His94
**AChE (4EY7)-Magnofluorine**	−9.5	Hydrogen bonding Hydrophobic interactions π-π stacked, π cation	Tyr124, Asp74 Trp86, Tyr337, His447 Tyr337, Phe338, Trp86
**BChE (6T9P)-Magnofluorine**	−9.8	Hydrogen bonding Hydrophobic interactions π-π stacked, π cation, π sigma	Gly116, Gly117, Ser198, Trp82 Phe329 Trp82, Phe329, His438, Trp231
**α-Gly (5NN8)-Magnofluorine**	−7.2	Hydrogen bonding Hydrophobic interactions π-π stacked, π anion	Arg600, Asp282 Trp481, Trp376, Phe525, Phe649 Trp481, Asp616

## Data Availability

Data are provided in a publicly accessible repository.

## Sample Availability

Samples of the compounds are not available from the authors.
